# Extracorporeal Membrane Oxygenation in Diffuse Alveolar Hemorrhage Secondary to Systemic Lupus Erythematosus

**DOI:** 10.14740/jocmr1685w

**Published:** 2014-02-06

**Authors:** Christine Pacheco Claudio, Emmanuel Charbonney, Madeleine Durand, Christophe Kolan, Mikhael Laskine

**Affiliations:** a Centre Hospitalier de l’Universite de Montreal, Montreal, Canada; b Hopital de Trois-Rivieres and Hopital du Sacre-Coeur de Montreal, Montreal, Canada; c Department of Medicine, Hopital Hotel-Dieu, CRCHUM, Montreal, Canada

**Keywords:** Extracorporeal membrane oxygenation, Diffuse alveolar hemorrhage, Systemic lupus erythematosus, Systemic anticoagulation, Continuous veno-venous hemodialysis, Bioavailability

## Abstract

Diffuse alveolar hemorrhage (DAH) is a rare and potentially deadly complication of systemic lupus erythematosus (SLE). We report two adult cases where extracorporeal membrane oxygenation (ECMO) was used as rescue therapy for severe respiratory failure in this setting. We discuss the risk related to coagulation disturbance and the need for the circuit anticoagulation in this particular setting. We also briefly discuss the clinical problem of lack of knowledge on the bioavailability of the immunosuppressive treatment with the use of ECMO. We think that ECMO should be used as rescue therapy in patients with DAH caused by SLE, but strategies for anticoagulation require further precision.

## Introduction

Extracorporeal membrane oxygenation (ECMO) is a circulatory and ventilatory support technology, used when perfusion or gas exchange disturbances, or both cannot be resolved with conventional treatments. It has been previously used in the context of diffuse alveolar hemorrhage (DAH) secondary to various vasculitis or auto-immune diseases.

Given the need of anticoagulation to prevent the formation of thrombus in the circuit, difficulties can be encountered in case of DAH, where preventing both thrombotic and hemorrhagic complications associated with ECMO becomes a challenge. In addition, patients diagnosed with DAH secondary to systemic lupus erythematosus (SLE) and other auto-immune diseases often present both an inflammatory state in addition to thrombocytopenia [1] and platelet dysfunction.

The use of ECMO as rescue therapy for refractory hypoxemia secondary to DAH caused by SLE has been reported in the pediatric population. Outcomes in one case series appear favorable despite the added risk of bleeding conferred by this type of hemodynamic support [2].

Its use in this particular clinical circumstance has only previously been described in one adult patient [3].

We report two adult cases where ECMO was used as rescue therapy for severe respiratory failure caused by DAH secondary to SLE. We discuss the risk related to coagulation disturbance and the need for the circuit anticoagulation, in this particular setting.

## Case Report

The first patient is a 33-year-old Haitian female previously diagnosed with SLE, manifested by serositis, vasculitis, proteinuria and positive ANA and anti-double stranded DNA titers. After the initial treatment by solumedrol pulses (1 g/day for 3 days), she was discharged on prednisone (1 mg per kg of body weight) and mycophenolate mofetil (1 g twice daily).

A month later, she presented to the Emergency Department with a principal complaint of progressive dyspnea, hemoptysis and diffuse abdominal pain.

Initial work-up demonstrated diffuse bilateral alveolar infiltrates on chest X-ray, and no evidence of any acute abdominal process on abdomen CT. Bronchoscopy was performed and confirmed alveolar hemorrhage. Blood cultures were positive for *Klebsiella pneumonaie*, and broad-spectrum antibiotic coverage was initiated.

The patient was then admitted to ICU for sepsis and hypoxemic respiratory insufficiency requiring mechanical ventilation in the setting of SLE flare-up. Over the next 24 h, the patient remained significantly hypoxemic despite conventional mechanical ventilation at maximal settings in pressure-controlled mode, with peak airway pressures of 50 cmH_2_O, positive end-expiratory pressure (PEEP) of 16 cmH_2_O, at a rate of 12 breaths/min with a FiO_2_ of 1.0. Despite various attempts to improve the oxygenation, her clinical deterioration required an alternate mode of oxygenation as rescue therapy.

Veno-venous ECMO was therefore initiated via, as usual in our institution, a heparin-coated 27 French double lumen Avalon^TM^ cannula placed in the right jugular vein. For the first 24 h, we used the trillium-coated Affinity NT filter (Medtronic^®^), then we changed it to heparin-coated Quadrox D filter (Maquet^®^) for the sake of effectiveness and long-term use. Systemic anticoagulation was not initiated because of the DAH. Initial output was 3.4 L/min and saturation measured on pulse oximetry rose quickly to over 94%. Pulse corticosteroids and intravenous cyclophosphamide were also initiated.

The patient then progressed into multi-organ failure with acute renal failure. Continuous veno-venous hemodialysis (CVVH) was required.

The CVVH filter was rinsed with heparin, but systemic heparin perfusion was not initiated with dialysis. After the CVVH was started, the alveolar hemorrhage progressed dramatically, at a rate of 200 mL/h. Coagulation studies were performed and revealed a prothrombin time of 72 sec. Despite correction of coagulation studies with fresh frozen plasma, cryoprecipitates and red blood cell transfusions, the patient died from profound hemodynamic collapse secondary to diffuse severe bleeding and septic shock 48 h later, which was confirmed by autopsy.

The second patient was a 36-year-old male, also of Haitian origin, known for diabetes and dyslipidemia. He was hospitalized for investigation of dyspnea and fatigue, and SLE was diagnosed on the basis of glomerulonephritis, positive ANA titer, alveolar infiltrates and arthritis. He received IV pulse solumedrol (1 g/day for 3 days) and was discharged after 9 days with 50 mg/day of oral prednisolone, with the plan to administer oral cyclophosphamide.

Two days following discharge, he was re-admitted for severe cellulitis and panniculitis of the right leg. Antibiotic treatment and intravenous corticosteroids were initiated. After surgical exploration, the patient was admitted to the ICU for severe sepsis, and then developed hypoxemic respiratory failure. Bronchoscopy revealed diffuse bleeding in both main stem bronchi. Despite conventional ventilator settings in the volume-controlled ventilation mode with PEEP of 12 cmH_2_O and FiO_2_ of 1.0, the patient remained significantly hypoxemic. Inhaled nitric oxide, then epoprostenol were initiated and the patient was placed on high frequency oscillatory ventilation (HFOV). Veno-venous ECMO was initiated 1 h later because the patient remained significantly hypoxemic and acidotic (SaO_2_ of 70%, PaO_2_ of 50 mmHg, arterial pH < 7.0).

Veno-venous ECMO was initiated via two heparin-coated cannulae installed in the left femoral vein (21 French) and in the right jugular vein (20 French). We used the same filter strategy as for the first patient. Initial output was 4.5 L/min and saturation measured on pulse oximetry rose quickly to 97%. Systemic anticoagulation with heparin was not initiated given the clinical diagnosis of DAH. Pulse intravenous corticosteroids were administered for 72 h, as was intravenous cyclophosphamide, in light of the significant clinical deterioration secondary to SLE flare-up. Few days later, he developed a thrombus in the ECMO circuit, and heparin was then initiated temporarily but soon stopped. No secondary bleeding was noted and there were no subsequent thrombotic complications. ECMO was continued for a total of 6 days.

Concomitantly, acute renal failure developed secondary to acute tubular necrosis exacerbating baseline nephritis secondary to SLE, which required dialysis for a total of 10 days. The CVVH filter was rinsed with heparin, but systemic heparin perfusion was not initiated with dialysis.

The patient recovered and was weaned off mechanical ventilation after 10 days. Creatinine returned to baseline. The patient was discharged from hospital on oral prednisone (60 mg daily) and intravenous cyclophosphamide. He was stable and free of neurologic sequelae at his last follow-up appointment 1 year later.

## Discussion

We report two cases of the use of ECMO in the setting of DAH secondary to SLE disease flare-up in adult population.

ECMO is generally used as rescue or bridge therapy, for cases where conventional ventilation and hemodynamic support measures have failed to improve the patient’s clinical condition. General indications for the use of ECMO are cardiac pump failure, secondary to cardiotomy or inability to wean cardiopulmonary bypass follow heart surgery, or heart failure, and respiratory insufficiency, more commonly secondary to acute respiratory distress syndrome (ARDS) [4], notably in the context of H1N1 epidemic [4, 5], pneumonia or graft dysfunction following lung transplant.

Formal indications for ECMO are severe hypoxemia with a PaO_2_/FiO_2_ of < 100 mmHg despite optimal ventilator settings or an alveolar arterial gradient of more than 600 mmHg in the absence of cardiogenic pulmonary edema or hypercapnia with a pH of less than 7.20 [6]. ECMO is contraindicated in cases of irreversible respiratory or cardiac disease, in patients over the age of 65 years old, or in patients who have been mechanically ventilated for over 5 days.

Both cases we describe had acute respiratory failure that did not respond to conventional support methods. Given their age and their potentially reversible underlying conditions (severe flare-up of SLE and systemic infection), they were appropriately considered for veno-venous ECMO.

Complications of ECMO include hemorrhage, thromboembolism, local infection and, less commonly, technical problems related to cannulation [6].

The first complication most often results from the need for systemic anticoagulation with heparin in order to avoid circuit thrombosis and systemic thromboembolism. For this reason, ECMO is often relatively contraindicated in patients at high risk of bleeding, notably trauma patients.

In both our patients, systemic anticoagulation was not used, as the risk of bleeding was deemed too high. Cannulae and ECMO circuit were coated with heparin and the filters with trillium or heparin directly by the manufacturer. In both patients, heparin was used to rinse the CVVH circuit. In the first case, this probably caused significant bleeding in the setting of septic shock.

Patients with DAH are particularly vulnerable to increased bleeding risk. In one case series of pediatric patients with DAH secondary to multiple baseline pathologies, patients were anticoagulated, albeit at slight lower clotting time levels, and did not have more hemorrhagic complications [2]. In the adult population, the initiation of ECMO without anticoagulation has been described [7]. The subsequent initiation of heparin caused profuse bleeding which was difficult to control, and required the use of aminocaproic acid [7]. In the case of SLE-induced DAH [3], systemic anticoagulation was initiated upon the installation of ECMO. No notable hemorrhagic complications were reported. Our first case illustrates how the use of heparin, albeit only to rinse the CVVH circuit, led to a significant increase in alveolar hemorrhage. It is the first case in the literature to our knowledge where the use of anticoagulation has aggravated DAH significantly. There were no thrombotic complications related to ECMO in this patient where heparin was not used. In our second patient, a thrombus did form in the circuit; however, there were no associated consequences.

Alternative measures to systemic anticoagulation in order to decrease the risk of clot formation in extracorporeal circuits exist. The use of regional citrate anticoagulation in critically ill patients at increased risk for bleeding requiring CVVH is a recognized alternative [8, 9]. This alternative to anticoagulation with an ECMO circuit has preliminarily been explored in animal studies [10] where no clot formation was noted in circuits after 24 h, but further studies are required in human subjects. Using anticoagulation with lower standard clotting time parameters in the pediatric population, as proposed by Kolovos et al [2], should equally be considered in adult patients with DAH. The use of ECMO without anticoagulation has also been described in trauma patients. Muellenbach et al [11] reported the prolonged use of ECMO without heparin as rescue therapy for ARDS in three patients with multiple injuries including traumatic brain injury, a contraindication to systemic anticoagulation. One of the three patients was diagnosed with thromboembolic disease in the inferior vena cava, appreciated on follow-up imaging after the discontinuation of ECMO and eventually required anticoagulation.

Another aspect of these two cases is the bioavailability of the immunosuppressive treatment with the use of ECMO as a supportive therapy. Indeed mainstay treatment of SLE flare-up with or without DAH consists of pulse IV corticosteroids [12] with the addition of immunosuppressive agents [13], notably cyclophosphamide, which appears to confer a better prognosis in a case-series of patients with SLE-induced DAH [14]. Unfortunately little is known about the bioavailability of the cyclophosphamide or other immunosuppressive agent in the setting of ECMO and whether dose adjustment is necessary.

The use of ECMO in adult patients diagnosed with DAH secondary to SLE has been previously described only once. In our two cases, the installation of veno-venous ECMO provided adequate gaseous exchanges and respiratory support while immunosuppressive agents became effective. Our second case of severe SLE flare-up was treated with solumedrol and cyclophosphamide and was successfully weaned off ECMO after 6 days with a return to baseline of both pulmonary and renal function including conserved functional class and cognitive function at long-term follow-up. Unfortunately, our first patient experienced incoercible bleeding following the rinsing of a dialysis circuit with heparin, which contributed to systemic hypoperfusion, multi-organ failure and death.

ECMO should be considered as rescue therapy in patients with severe hypoxemic respiratory failure secondary to DAH caused by SLE without use of systemic anticoagulation therapy. Our cases illustrate that withholding it did not cause significant thrombotic complications.

Further studies are needed to determine whether ECMO as rescue therapy confers better survival outcome in patients diagnosed with DAH secondary to SLE, and strategies for anticoagulation require further precision. Investigations will, however, be limited by the rarity of this medical condition.
